# Idiopathic Exposed Bone Lesions of the Jaw

**DOI:** 10.3390/dj7020055

**Published:** 2019-06-01

**Authors:** Božana Lončar Brzak, Vanja Vučičević Boras, Ana Kotarac Knežević, Mato Sušić, Sven Seiwerth, Dragana Gabrić

**Affiliations:** 1 Department of Oral Medicine, School of Dental Medicine, University of Zagreb, Zagreb 10 000, Croatia; borasvanja@yahoo.com; 2 Department of Oral Surgery, School of Dental Medicine, University of Zagreb, Zagreb 10 0000, Croatia; akotarac@sfzg.hr (K.K.A.); susic@sfzg.hr (S.M.); dgabric@sfzg.hr (G.D.); 3 Department of Pathology, School of Medicine, University of Zagreb, Clinical Hospital Centre Zagreb, Zagreb 10 000, Croatia; sven.seiwerth@mef.hr

**Keywords:** osteonecrosis, jaw, oral cavity, therapy

## Abstract

Introduction: Osteonecrosis of the jaw is defined as exposed bone in the oral cavity that does not heal longer than eight weeks after identification. The two most common predisposing factors for osteonecrosis of the jaw are medication-related and radiotherapy. Rarely, exposed bone in the maxillofacial region can occur due to other causes and represents a clinical and therapeutic challenge for the dentist because there is no universally accepted treatment protocol. Case presentation: We report a case of a patient with two idiopathic lesions of exposed bone which have healed after systemic antibiotic therapy, seven weeks after the first examination. Conclusion: Exposed bone lesions of the jaw are a rare entity and are poorly documented in the literature. It is necessary to exclude possible local or systemic contributing factors. Surgical and conservative therapy (antibiotics) are the treatment of choice.

## 1. Introduction

Osteonecrosis of the jaw (ONJ) is defined as exposed bone in the oral cavity that does not heal longer than eight weeks after identification [1]. Most of the reported cases are related to medications (intravenous and oral bisphosphonates, denosumab, and bevacizumab) or previous head and neck radiotherapy [1,2]. The mandible, especially its lingual side, is predominantly affected because of poorer blood supply. Rarely, exposed bone in the oral cavity can occur due to other causes which are mostly recognized through case reports. 

The aim of this case report was to describe a rare case of idiopathic mandibular exposed bone lesions in the patient without known predisposing factors. The patient gave her informed consent for using and publishing her medical data and photographs for scientific purposes.

## 2. Case Presentation

A 77-year old female came to the department of oral medicine complaining about an ulceration on the left side of her tongue. A few days earlier, she had her lower left first molar non-surgically extracted. She reported that the tooth was very sharp and irritated her tongue. Now she was edentulous in the upper and lower jaw. Her medical history revealed hypertension and a penicillin allergy. She was taking nebivolol, lysinopril, and acetylsalicylic acid daily, and pantoprazolum occasionally. She did not smoke or consume alcohol. Clinical examination revealed an oblong ulceration on her left side of the tongue, the size of 1.5 × 4 cm. The extraction wound was healing fine. The patient was given a perilesional instillation of corticosteroid (methylprednisolone, 0.5 mL of 40 mg/mL,) around the tongue wound and local therapy, which included an antiseptic solution (chlorhexidine gluconate 2%) and corticosteroid ointment (betamethasone in orabase) to be applied four times a day. 

**Figure 1:**
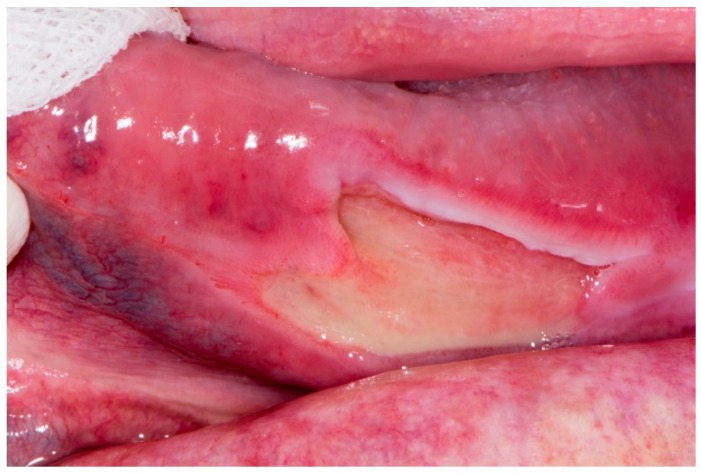
Tongue ulceration.

**Figure 2:**
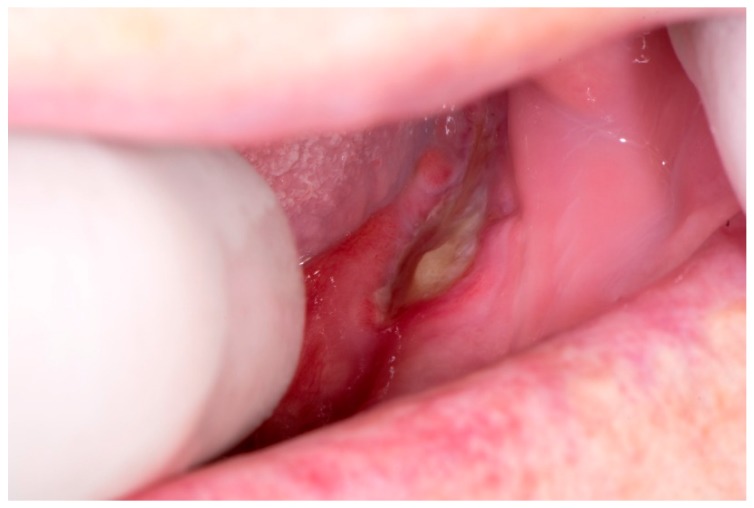
Exposed bone in distal part of the mandible.

**Figure 3:**
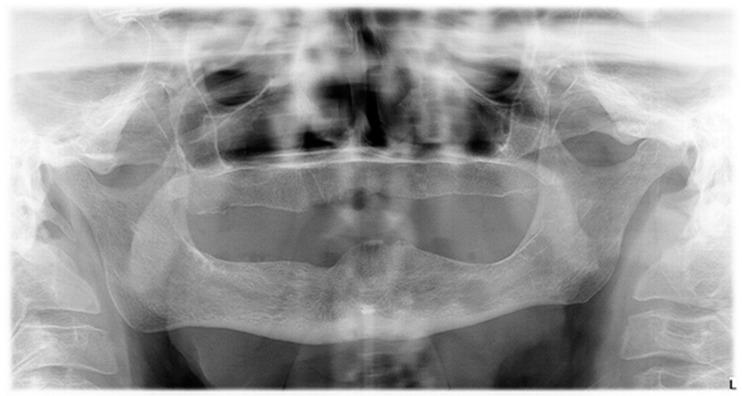
Orthopantomograph.

At the control examination, after one week, the tongue lesion was enlarged (Figure 1). The extraction wound healed, but an exposed area of bone was visible on the lingual side of the lower jaw, in the projection of the wound (Figure 2). The patient did not wear dentures after the tooth extraction, so this was not a denture pressure sore, and also, this was not trauma from tooth extraction because it appeared a week after in the distal part of the mandible. Conventional radiographic analysis (orthopantomograph) had shown a healthy bone without osteolysis in that area (Figure 3). An oral surgeon specialist smoothed the visible bone edges with a chisel without elevating the mucoperiosteal flap (to avoid ischaemia and further spreading of the wound). There was no bone sequestration and the exposed bone was not removed and histopathologically analyzed. The patient received local therapy, which included antiseptic solution (chlorhexidine gluconate 2%) and corticosteroid ointment (betamethasone in orabase) to be applied four times a day.

**Figure 4:**
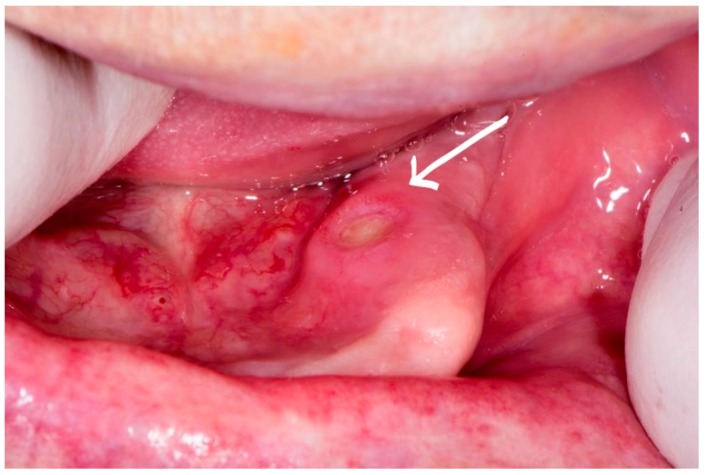
Exposed bone in anterior part of the mandible.

At the next control examination after one more week, the earlier lesions remained unchanged, but another area of exposed bone appeared in the edentulous ridge of the same jaw in the anterior region, and also on the lingual side of the jaw, with a diameter of 0.5 cm (Figure 4). The sharp and protruding edges of the first lesion of exposed bone in the distal part of the mandible were manually reduced one more time, again without elevation of the mucoperiosteal flap. The patient was referred to make a complete blood count, blood glucose, and serum iron level to exclude anemia or diabetes mellitus as possible causes of slow healing, and all findings were within a normal range. The patient received local therapy for home, according to the previous protocol.

The control examination was after one week and there were no changes in the clinical appearance of the lesions. Since there was no improvement despite local therapy, a biopsy sample of mucosa was taken from the tongue ulceration to exclude possible malignant transformation. The histopathological finding was: “On most of the surface of the received sample, there is no preserved surface epithelium and there is a granulation tissue. At the edge of the sample, there is a multilayer squamous epithelium, which is degeneratively and reparatory transformed. The finding corresponds to the edges of an ulcer. There is no tumor tissue in the examined and received material”.

We decided to try the conservative treatment and the patient was then prescribed systemic antibiotic therapy of clindamycin 3 × 600 mg and metronidazole 3 × 400 mg for ten days.

At the control examination 7 weeks after the first clinical examination and 10 days after antibiotics were prescribed, all lesions healed.

## 3. Discussion

Exposed bone in the maxillofacial region represents a clinical and therapeutic challenge for the dentist. Pathophysiology of the development of exposed bone lesions in patients without predisposing factors is not yet clarified. In cases where osteonecrosis develops in the absence of osteonecrosis-related medications, it can be described as “oral ulceration and bone sequestration” (OUBS) and has been reported in the literature under various names [2]. It is considered that bone sequestration develops after bone exposure, due to disrupted vascular supply and possible subsequent infections [2]. The predilectional localization for this type of lesion is posterior lingual mandibular bone, just as it was in our case. Although the bone was healthy in our patient and we did not confirm bone necrosis radiographically, it is possible that our lesion and OUBS share the same etiology. The incidence and prevalence of OUBS in the general population is not known. Also, in early stages of the disease, decalcification is limited, and changes are not visible by plain radiography [3]. 

Possible etiologic factors for exposed bone lesions were reported in the literature and consist of trauma, odontogenic infections, herpes zoster infection associated osteonecrosis, HIV-associated necrotizing ulcerative periodontitis, benign sequestration of the lingual plate [1], long-term corticosteroid usage [4], ischaemia, occlusion, and coagulopathies [5,6,7]. 

In our case, we presume that vascular ischaemia after local anesthesia for tooth extraction has caused necrosis of the mucosa with bone exposure in the lingual distal part of the mandible. There is also no clear explanation for the development of the second lesion of exposed bone, in the anterior part of the lingual aspect of the mandible. A local trauma during reducing the sharp edges of the first lesion of exposed bone might have been the trigger for its development, and it was also on thin and poor in connective tissue oral mucosa.

There are no universally accepted treatment protocols for exposed bone in the maxillofacial region. The treatment can be conservative or surgical [2]. Surgical management is usually a late treatment option [8,9]. Conservative therapy implies local antiseptic and topical antibiotic therapy and systemic antibiotic therapy, if necessary [10,11,12]. In our case, reducing the sharp bone edges to exclude further local trauma and local antiseptic and anti-inflammatory therapy has not been beneficial, as the lesions persisted, and another new area of exposed bone appeared in the mouth. Systemic antibiotic therapy has led to a complete resolution of all lesions, both on the tongue and on the mandible. 

## 4. Conclusions

Exposed bone lesions in the jaw are a rare entity and are poorly documented in the literature, therefore they represent a therapeutic challenge. It is necessary to exclude possible local or systemic contributing factors. Surgical and conservative therapy (antibiotics) are the treatment of choice. 

## References

[1] Khosla S., Burr D., Cauley J., Dempster D.W., Ebeling P.R., Felsenberg D., Gagel R.F., Gilsanz V., Guise T., Koka S. (2007). American Society for Bone and Mineral Research. Bisphosphonate-Associated Osteonecrosis of the Jaw: Report of a Task Force of the American Society for Bone and Mineral Research. J. Bone Miner. Res..

[2] Khan A.A., Morrison A., Hanley D.A., Felsenberg D., McCauley L.K., O’Ryan F., Reid I.R., Ruggiero S.L., Taguchi A., Tetradis S. (2015). Diagnosis and Management of Osteonecrosis of the Jaw: A Systematic Review and International Consensus. J. Bone Miner. Res..

[3] Hutchinson M., O’Ryan F., Chavez V., Lathon P.V., Sanchez G., Hatcher D.C., Indresano A.T., Lo J.C. (2010). Radiographic Fndings in Bisphosphonate-Treated Patients with Stage 0 Disease in the Absence of Bone Exposure. J. Oral Maxillofac. Surg..

[4] Assouline-Dayan Y., Chang C., Greenspan A., Shoenfeld Y., Gershwin M.E. (2002). Pathogenesis and Natural History of Osteonecrosis. Semin. Arthritis Rheum..

[5] Jones J.P. (1997). Coagulopathies in the Pathogenesis of Osteonecrosis. Orthop. Trauma.

[6] Jarman M.I., Lee K., Kanevsky A., Min S., Schlam I., Mahida C., Huda A., Milgrom A., Goldenberg N., Glueck C.J. (2017). Case Report: Primary Osteonecrosis Associated with Thrombophilia-Hypofibrinolysis and Worsened by Testosterone Therapy. BMC Hematol..

[7] Henien M., Patel V., Sproat C., McGurk N. (2016). Spontaneous Osteonecrosis of the Maxilla. Dent. Update.

[8] Wilde F., Heufelder M., Winter K., Hendricks J., Frerich B., Schramm A., Hemprich A. (2011). The Role of Surgical Therapy in the Management of Intravenous Bisphosphonates-Related Osteonecrosis of the Jaw. Oral Surg. Oral Med. Oral Pathol. Oral Radiol. Endod..

[9] Mücke T., Koschinski J., Deppe H., Wagenpfeil S., Pautke C., Mitchell D.A., Wolff K.D., Hölzle F. (2011). Outcome of Treatment and Parameters Influencing Recurrence in Patients with Bisphosphonate-Related Osteonecrosis of the Jaws. J. Cancer Res. Clin. Oncol..

[10] Khan A.A., Sandor G.K., Dore E., Morrison A.D., Alsahli M., Amin F., Peters E., Hanley D.A., Chaudry S.R., Dempster D.W. (2009). Bisphosphonate Associated Osteonecrosis of the Jaw. J. Rheumatol..

[11] Khan A.A., Sandor G.K., Dore E., Morrison A.D., Alsahli M., Amin F., Peters E., Hanley D.A., Chaudry S.R., Dempster D.W. (2008). Canadian Consensus Practice Guidelines for Bisphosphonate Associated Osteonecrosis of the Jaw. J. Rheumatol..

[12] Ruggiero S.L., Dodson T.B., Assael L.A., Landesberg R., Marx R.E., Mehrotra B. (2009). American Association of Oral Maxillofacial Surgeons Position Paper on Bisphosphonate-Related Osteonecrosis of the Jaw—2009 Update. Aust. Endod. J..

